# Decreased severity of experimental autoimmune arthritis in peptidylarginine deiminase type 4 knockout mice

**DOI:** 10.1186/s12891-016-1055-2

**Published:** 2016-05-05

**Authors:** Akari Suzuki, Yuta Kochi, Hirofumi Shoda, Yu Seri, Keishi Fujio, Tetsuji Sawada, Ryo Yamada, Kazuhiko Yamamoto

**Affiliations:** Laboratory for Autoimmune Diseases, Center for Integrative Medical Sciences, The Institute of Physical and Chemical Research (RIKEN), 1-7-22 Suehirocho, Tsurumi-ku, Yokohama City, Kanagawa 230-0045 Japan; Department of Allergy and Rheumatology, Graduate School of Medicine, the University, 7-3-1 Hongo, Bunkyo-ku, Tokyo, 113-8655 Japan; Department of Joint Disease and Rheumatism, Tokyo Medical University, 6-1-1 Shinjuku, Shinjuku-ku, Tokyo, 160-8402 Japan; Center for Genomic Medicine, Kyoto University, Yoshida-konoe-cho, Sakyo-ku, Kyoto, 606-8501 Japan

**Keywords:** Peptidylarginine deiminase type 4, Rheumatoid arthritis, Collagen-induced arthritis mice, TNF-α, Citrullination

## Abstract

**Background:**

Peptidylarginine deiminase type 4 (PADI4) has been identified as a susceptibility gene for rheumatoid arthritis (RA) by genome-wide association studies. PADI4 is highly expressed in the bone marrow, macrophages, neutrophils, and monocytes. Peptidyl citrulline is an interesting molecule in RA because it is a target antigen for anti-citrullinated peptide antibodies, and only PADs (translated proteins from PADI genes) can provide peptidyl citrulline via the modification of protein substrates. The aim of this study was to evaluate the importance of the PADI4 gene in the progression of RA.

**Methods:**

We generated Padi4 knockout (Padi4^−/−^) DBA1J mice. The Padi4^−/−^ DBA1J and wild-type mice were immunized with bovine type II collagen (CII) to develop collagen-induced arthritis (CIA). The expression of various inflammatory cytokines and Padi genes in immune cells was detected by the real-time TaqMan assay. Cytokine concentrations in sera were measured by enzyme-linked immunosorbent assays. Localization of the PAD4 and PAD2 proteins was indicated by immunohistochemistry.

**Results:**

We demonstrated that the clinical disease score was significantly decreased in the Padi4^−/−^ mice and Padi4 expression was induced by CII immunization. In the Padi4^−/−^ mice, serum anti-type II collagen (CII) immunoglobulin M (IgM), IgG, and inflammatory cytokine levels were significantly decreased compared with those in the wild-type mice. Padi2 expression was induced in the immune cells of the Padi4^−/−^ mice as a compensation for the defect in Padi4.

**Conclusions:**

Padi4 affected disease severity in the CIA mice and was involved in the enhancement of the collagen-initiated inflammatory responses.

**Electronic supplementary material:**

The online version of this article (doi:10.1186/s12891-016-1055-2) contains supplementary material, which is available to authorized users.

## Background

Autoimmune diseases are caused by multiple factors, including genes and environmental factors. Rheumatoid arthritis (RA) is one of the most common systemic autoimmune diseases in humans, with a worldwide prevalence of approximately 1 % [1, 2]. It is characterized by inflammation of the synovial tissues and the formation of a rheumatoid pannus, which is capable of eroding the adjacent cartilage and bone and causing subsequent joint destruction.

Many non-HLA loci have been identified as RA susceptibility genes in genome-wide association studies (GWASs). We previously identified peptidylarginine deiminase type 4 (PADI4) as an RA susceptibility gene in a large-scale, case-control association study using a gene-based GWAS method [1]. We identified single nucleotide polymorphisms (SNPs) associated with the development of RA in the coding region of PADI4, and these RA-causal SNPs were associated with allelic imbalances in gene expression, with pathological relevance. PADI4 susceptibility to RA was initially observed only in Asian populations [3–7]. However, PADI4 has also been associated with RA in mega-GWASs involving multiple ethnic groups [8–11]. Therefore, PADI4 has been recognized as a common genetic risk factor for RA.

Although some of the risk genes identified by GWASs are shared among autoimmune diseases, PADI4 has been associated only with RA [12, 13]. The reason for this specificity may be that PADs have catalytic activity in the conversion of peptidylarginine to peptidyl citrulline, and citrulline-containing epitopes are the most specific targets of RA-specific autoantibodies, well known as anti-citrullinated protein antibodies (ACPA), e.g., cyclic citrullinated peptide (CCP) antibody. The strongest genetic association in RA was observed in the HLA region on chromosome 6p21. This region extends over 3.6 Mb, including the major histocompatibility complex (MHC)-class I, II, and III molecules, and contains many genes with immunoregulatory functions. Previously, it was reported that HLA-DRB1 SE alleles were associated with ACPA-positive RA but not with ACPA-negative RA [14, 15]. Therefore, it appears that PADI genes functionally interact with the HLA-DRB1 SE allele and are related to the production of ACPA. Ethnic heterogeneity in the genetic risk factors for RA, including genetic variations and effect sizes such as those for the HLA-DRB1 alleles, protein tyrosine phosphatase, non-receptor type 22 (PTPN22) alleles, and PADI4 alleles, has been noted in different populations. The difference in the contribution of PADI4 to RA between Asians and Europeans may be caused by the difference in the prevalence of smoking as well as other genetic and environmental factors among these ethnic groups [16–18]. The interactions among smoking history, PADI4 polymorphisms, and HLA-DRB1 SE have been investigated in some populations [16, 17, 19]. The reasons for the different effect sizes of the other susceptibility genes remain unclear; moreover, little or nothing is known about the manner in which these genetic risks pathologically affect the mechanisms underlying RA development. PAD expression and activity are increased in the lung of a smoker and also seropositivity, e.g., RF associated with smoking status [20]. HLA-DRB1 SE alleles conferred the highest risk of developing anti-CCP antibodies [18] and PADI4 and HLA-DRB1 SE had also associated with joint destruction and anti-CCP positivity [21]. However, directly functional association between PADI4 and HLA-DRB1 SE has been not shown.

Many citrullinated proteins/peptides have been reported, such as filaggrin, K1 keratin, and fibrinogen [22–30]. Dermal citrullination seems to be the most thoroughly investigated. Originally, the antigen of the circulated citrullinated peptide antibody was based on a citrullinated filaggrin peptide. These citrullinated proteins are only provided during post-translation using PADIs because citrulline is a noncoding amino acid. Furthermore, biological events such as inflammation, apoptosis, and aging increase post-translational citrullination [31–33]. Citrullinated proteins are candidate autoantigens; however, their pathophysiological functions in RA development remain unknown.

The function of PAD4, which is encoded by the PADI4 gene, plays a role in the intranuclear citrullination of histones and regulation of gene expression [34, 35]. The citrullination of histones is apparently linked to histone methylation and acetylation in the regulatory mechanism of gene expression. In particular, citrullination of the histone H4R3 related to the p53 pathway plays an important role in response to DNA damage in carcinogenesis [36]. PAD4 also modified ELK1, a major transcriptional element, and citrullinated ELK1 activated the expression of the c-Fos oncogene [37]. Furthermore, PADI2, a member of the PADI gene family, is not localized in the nucleus; however, PAD2 affects gene transcription via the citrullination of IKK-gamma [38]. These data indicate that citrullination plays an important role in transcription. Therefore, PADI4 is genetically and functionally important for both RA and carcinogenesis.

As a result, the PAD4 enzyme became a candidate target for RA therapy [39]. It was reported that Cl-amidine, which is one of the pan-PAD inhibitors, suppressed arthritis induced by collagen-induced arthritis (CIA) [40]. Cl-amidine also strongly affected breast tumor progression by inhibiting PADIs [41].

To investigate the function of PADI4 in physiological conditions and the relationship between citrullination and RA development, we analyzed mice that lacked the PAD4 enzyme and generated Padi4^−/−^ DBA1J mice by the speed congenic method. We found that PAD4 affected the development and progression of CIA in an animal model of RA. However, the incidence was not significantly different. In addition, serum anti-Type II collagen (CII), IgM, IgG, Il-6, and tumor necrosis factor alpha (TNF-α) levels in the Padi4 knockout mice with CIA were significantly decreased compared with those in the wild-type mice with CIA.

## Methods

### Mice

Congenic inbred Padi4^−/−^ C57BL/6 J (B6) background mice [36] were backcrossed with DBA1JNCrIj (DBA1J) (>99.5 % of the DBA1J) by the speed congenic method based on the protocol provided by Applied Biosystems (http://tools.invitrogen.com/content/sfs/manuals/cms_039321.pdf). The DBA1J background was confirmed by genotyping 269 single nucleotide polymorphisms (SNPs) using the Invader assay. Southern blot analyses and genotypes were confirmed by PCR analyses to verify correct targeting and transmission of the genotypes. The primer sequences for PCR genotyping were Padi4_F5: CAGTGGGTCAGTGACTGTC, Padi4_R5: CGAGAGCTAGCCTGGGATC, Neo_F1: CAGCTGTGCTCGACGTTGTC, Neo_R1: CAACGCTATGTCCTGATAGC, beta-actin (Actb)_F1: CTCTATCACTGGGCATCGAG, and Actb_R1: GCAAGCTCCGCCTACACTG. We used Southern blotting and reverse transcription-polymerase chain reaction (RT-PCR) to determine the genomic structure and presence of the Padi4 transcripts [36]. DBA1JNCrIj (DBA1J) mice were purchased from Charles River Japan. All mice were maintained under specific pathogen-free conditions. The local ethics committee of Animal Experiments in the RIKEN IMS approved all animal procedures.

### Induction of CIA and assessment of arthritis

Male, wild-type DBA1J or Padi4^−/−^ DBA1J mice aged 8–10 weeks were immunized on day 0 with complete Freund’s adjuvant (CFA, Chondrex, Redmond, WA) containing 100 μg of bovine type II collagen (Chondrex) in a 1:1 mixture of adjuvant and collagen. The injections were subcutaneously administered into the base of the tail [42]. The control mice received the same volume of reagent without the collagen. On day 21, the mice were administered an intradermal booster injection with a mixture of incomplete Freund’s adjuvant (IFA, Chondrex) and collagen near the former injection sites. Arthritis was assessed using the following clinical scoring system for each limb: 0, normal; 1, redness and/or swelling in one joint; 2, redness and/or swelling in more than one joint; 3, redness and/or swelling in the entire paw; and 4, deformity and/or ankylosis. The maximum score was 16 per mouse.

### Detection of anti-CII antibodies

Serum samples were obtained from all experimental animals prior to immunization and on days 21, 28, 35, 42, 49, and 56 after the first immunization. Anti-CII antibodies were detected by ELISA at a dilution of 1:8000 [43]. Polystyrene 96-well microtiter plates (AGC Techno Glass, Tokyo, Japan) were coated with 10 μg/mL of bovine type II collagen in 5-mM acetic acid overnight at 4 °C. The plates were then washed, and nonspecific binding was blocked using the blocking buffer (10 % albumin, bovine, F-V, pH 5.2/T-PBS, pH 7.5) for 2 h at room temperature (RT). Following extensive washing, all subsequent dilutions were made in sample dilution buffer (0.5 % albumin, bovine in T-PBS) for 2 h at RT. The plates were washed and incubated with anti-mouse IgG (Fab)2 (1:2000, Invitrogen, Carlsbad, CA), anti-mouse IgM (Fab)2 HRP-conjugated (1:5000, MBL, Tokyo, Japan), anti-mouse IgG1 (1:2000, Invitrogen), anti-mouse IgG2a (1:2000, Invitrogen), anti-mouse IgG2b (Invitrogen), and anti-mouse IgG3 antibodies, respectively, for 1 h at RT. After washing, the binding of anti-CII antibodies was revealed by color development with OPD (Sigma-Aldrich, St. Louis, MO). The plates were then read on a microplate reader (Bio-rad, Hercules, CA) at a wavelength of 450 nm. Pooled polyclonal mouse serum with a known anti-CII antibody was included in each test plate and used as a positive control and standard.

### Isolation of cells from spleen

The DBA1J mice splenocytes were washed with MACS wash buffer (0.5 % ovine serum albumin, 2-mM ethylenediaminetetraacetic acid in PBS) before resuspension at 1 × 10^8^ cells/mL for magnetic separation. For the isolation of granulocytes, dendritic cells, T-cells, B-cells, and macrophages, microbeads or biotin conjugated with anti-Gr-1, anti-MHC-class II, anti-CD5, anti-CD45R, and anti-CD11b antibodies and a magnetic column separation system (Miltenyi Biotech, Bergisch Gladbach, Germany) were used according to the manufacturer’s instructions. Purified cells were used for real-time Taqman expression analysis and microarray analysis. For the culture of macrophages and granulocytes, isolated CD11b-positive cells and Gr-1-positive cells were used for the cytokine assay after lipopolysaccharide stimulation.

### Bone marrow macrophage (BMM) cultures

BMMs were obtained as previously described [44, 45]. Briefly, femurs from the wild-type and Padi4^−/−^ mice were flushed with 2 mL of Dulbecco’s modified Eagle’s medium (DMEM, Sigma) using a 26-gauge needle. The bone marrow plugs were centrifuged and resuspended in ice-cold complete media (DMEM, 10 % fetal bovine serum, 1 % penicillin streptomycin, 1 % glutamine, and 0.5-uM β-mercaptoethanol). Precursor cells were generated by culture in the presence of 1-nM interleukin (IL)-3 (Miltenyi Biotech) and 0.44-nM macrophage colony-stimulating factor (Miltenyi Biotech) for 24 h at 37 °C. Then, the cells were differentiated into adherent macrophages by 3 days of culture without IL-3 in complete media. The cultured BMMs were used for RNA isolation.

### RNA isolation and real-time TaqMan RT-PCR analysis for gene expression

RNA was extracted from isolated cells using an RNAeasy mini kit (QIAGEN, Venlo Park, The Netherlands), and each RNA sample was treated with DNaseI (QIAGEN) according to the manufacturer’s instructions. The total RNA was reverse-transcribed to cDNA using a high-capacity cDNA reverse transcription kit (Life Technologies Inc., Carlsbad, CA) in a 96-well format. The housekeeping gene mouse GAPD was chosen as an endogenous control for the normalization of expression data against each gene. All PCR primers and TaqMan probes were designed and purchased from Life Technologies according to the company’s protocols. Briefly, the real-time PCR reaction was performed at 95 °C for 10 min, followed by 40 cycles of template denaturation at 95 °C for 15 s, with an annealing temperature of 60 °C for 1 min. The relative transcription levels of the individual target genes were normalized using the internal control. All data were analyzed using PE Applied Systems sequence Detector 2.2 software (Applied Biosystems, Carlsbad, CA). The comparative threshold cycle method was used to quantify data. The results represent the expression level of the target gene relative to that of GAPD by defining ΔΔCt.

### ELISA for cytokines

Cytokine levels in the sera and supernatant of cell cultures were measured using the Ready-SET-GO ELISA set for TNF-α, IL-1β, and granulocyte-macrophage colony-stimulating factor (GM-CSF) (eBioscience, San Diego, CA) in accordance with the manufacturer’s instructions. Briefly, polystyrene 96-well microtiter plates (AGC Techno Glass) were coated with capture antibody, and blocking reactions were performed after washing the plates. Then, the diluted and standard samples were added to each well. After detection of the antibody reaction, TMB solution as a substrate was incubated in each well, and the reaction was stopped using 10 % H_2_SO_4_. Detection limits were 8–1000 pg/mL for TNF-α and Il-1β ELISA and 4–500 pg/mL for IL-6 and GM-CSF ELISA.

### Histological staining

Paraffin-embedded sections of mouse knee joints and spleens were provided by Genostaff (Tokyo, Japan). Briefly, the spleens were isolated and fixed in phosphate-buffered 4 % paraformaldehyde and embedded in paraffin wax (Wako, Tokyo, Japan). Whole knee joints were collected from the sacrificed mice and fixed, decalcified, embedded in paraffin, sectioned, and subjected to histological analysis. The tissue sections (6 μm) were heated at 95 °C for 10 min in a target retrieval solution (Dako, Glostup, Denmark) for antigen recovery. Then, the sections were incubated with 3 % H_2_O_2_ in methanol for inactivation of the endogenous peroxidase at RT for 30 min. The sections were washed with PBS buffer and incubated with primary antibodies. The immunostained sections were processed using the VECTASTAINABC kit and VECTOR M.O.M Immunodetection kit (Vector Labs, Burlingame, CA). Rabbit anti- CCP (1:1000, Abbiotec, San Diego, CA), rabbit anti-mouse PAD4 (1:1000, Novus Biologicals, Littleton, CO), and mouse anti-citrullinated fibrinogen antibodies (1:1000, ModiQuest, Noord-Brabant, The Netherlands) were used as primary antibodies. The immunoreactive signals were visualized using the DBA substrate (Vector Labs), which stains the target protein brown. Hematoxylin (Wako) was used for counterstaining.

For immunofluorescence double staining, rat anti-mouse CD3-conjugated Alexa Fluor® 647 (1:25, AbD serotec, Hercules, CA), rat anti-mouse CD56-conjugated Alexa Fluor® 647 (1:75, Oncogene Research Products, San Diego, CA), rat anti-mouse CD45R (anti-B220)-conjugated Cy5 (1:200, Cell Marque, Rocklin, CA), rat anti-mouse F4/80 (1:200, eBioscience), and rat anti-mouse Ly6/G (1:100, BD, Franklin Lakes, NJ) labeled with Alexa Fluor® 647 were used as the primary antibodies. PAD4 (1:25) and PAD2 (1:25, Proteintech, Chicago, IL) were detected using secondary antibodies labeled with Alexa Fluor® 546. To eliminate nonspecific staining caused by Fc receptor staining of the lymphoid and hematopoietic cells, an Fc receptor blocker (Innovex Biosciences; Richmond, CA) was used. For counterstaining, 4’, 6-diamidino-2-phenylindole Vectashield (Vector Labs) was used. For negative controls, Alexa Fluor 647 goat anti-rat IgG (H + L) conjugate and Alexa Fluor 546 goat anti-rabbit IgG (H + L) conjugate (Invitrogen) were used. Immunofluorescence signals were examined using an Olympus photomicroscope (Tokyo, Japan). Informed consent was obtained from each subject and the study was performed in compliance with the ethical guidelines of the RIKEN.

### Statistical analysis

All statistical analyses were performed using Excel (Microsoft, Redmond, WA).

## Results

### CIA development in the Padi4 knockout mice

The Padi4^−/−^ mutant mice were created as previously described [36]; we confirmed the genomic construct by Southern blotting and the absence of Padi4 expression in the knockout mice by RT-PCR (Additional file 1). The knockout animals were back-crossed to the DBA1J mice until they contained >99.5 % of the DBA1J genome. Histological analysis of the immune tissues by hematoxylin–eosin staining indicated that there was no difference; however, a slight difference among the genotypes was observed in the skin keratin layer (Additional file 1E). The life span of Padi4KO mice was comparable to wild-type mice, and that those mice did not develop spontaneous disease.

**Fig. 1:**
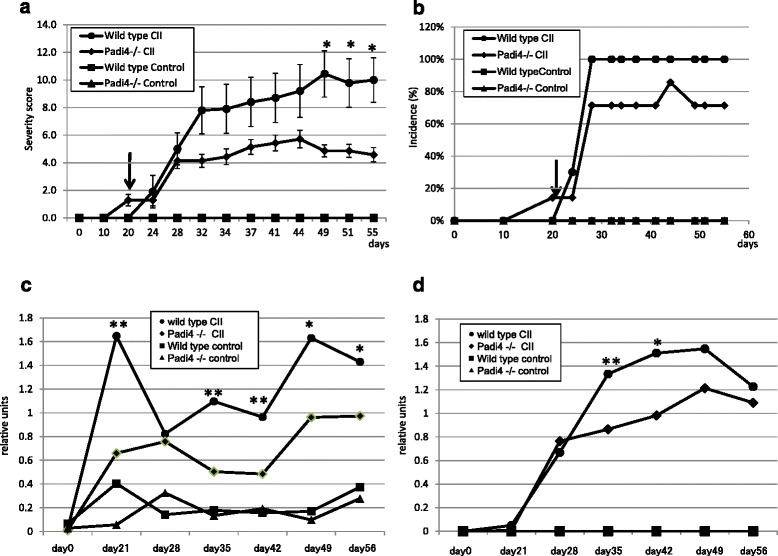
Comparative analyses of Padi4^−/−^ and wild-type mouse models of experimental arthritis. Wild-type (*n* = 10) and Padi4^−/−^ (*n* = 7) mice were immunized with bovine type II collagen (CII) and boosted on day 21 (arrowheads). As control mice, Wild-type (*n* = 5), and Padi4^−/−^ mice (*n* = 5) were immunized with PBS. **a** Arthritis severity score. The severity of arthritis was graded according to the scale defined in Materials and Methods. The data are presented as the mean clinical score ± SEM. *P < 0.05 (*U*-test). **b** Incidence of arthritis. The percentage of affected animals in each group was determined on each day. The IgM isotype (c) and IgG isotype (d) of anti-CII antibodies in the wild-type and Padi4^−/−^ mice sera before and after injection, as determined by ELISA. * *P* < 0.05, **P < 0.01 (Student’s *t*-test)

CIA was significantly less severe in the Padi4^−/−^ mice than in the wild-type controls (*p < 0.05, *U*-test, Fig. 1a). However, there was no significant difference in the incidence of CIA (Fig. 1b). To clarify whether the Padi4 gene controls susceptibility to CIA, all immunized mice-generated circulating anti-CII antibodies were analyzed by ELISA. The anti-CII IgG and IgM titers in the wild-type CIA mice sera were compared with those in the Padi4^−/−^ CIA mice sera. The anti-CII antibody titers were significantly decreased in the sera of the Padi4^−/−^ mice (Fig. 1c, d). With regard to the subsets of anti-CII IgG, there was no difference in IgG2b and IgG3 levels on days 35 and 42 (data not shown). However, anti-CII IgG1 and IgG2a levels were significantly lower in the sera of the Padi4^−/−^ mice than in that of the wild-type mice (Additional file 2).

### Expression patterns of the PADI genes

**Fig. 2:**
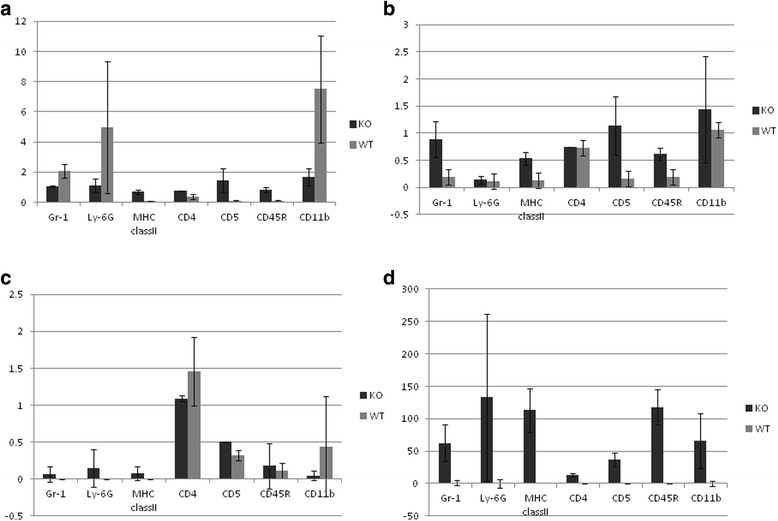
Expression of Padi genes in immune cells. Data are obtained from wild-type mice (*n* = 3) and Padi4^−/−^ mice (*n* = 3) and are presented as relative units against GAPDH. **a** Padi4, **b** Padi2, **c** Padi3, and **d** Padi6. Values represent the means ± standard deviations of data for each sample

We also investigated the Padi2 and Padi4 expression patterns in the mouse spleen using immunofluorescence histochemistry (Additional file 3). PAD4+ cells partially merged with CD3+ T-cells, CD45R (B220) + B-cells, and Ly-6G+ granulocyte cells, and they primarily merged with F4/80+ macrophage cells and CD56+ NK cells. In F4/80+ macrophages, PAD4 was localized to nuclear and perinuclear locations. PAD2+ cells were also partially merged with T-cells (CD3+ cells), B-cells [CD45R (B220) + cells], macrophages (F4/80+ cells), NK cells (CD56+ cells), and granulocytes (Ly-6G+ cells). Therefore, PAD2 and PAD4 were widely expressed in the immune cells. These data were consistent with the expression pattern in human blood [32, 46]. Padi4 mRNA was highly expressed in granulocytes (Gr-1+ cells) (Fig. 2a, granulocyte); however, PAD4+ cells rarely merged with granulocytes (Ly-6G+ cells) (Additional file 3). This result contradicts those of previous studies in human cells [32, 47].

A family of five Padi genes is clustered on chromosome 1p35-36 and chromosome 4D3-4E1 in humans and mice, respectively. To investigate whether the Padi4 gene controls other Padi genes, we performed real-time RT-PCR Taqman with cells from subsets of Padi4^−/−^ splenocytes. The mouse Padi4 gene is primarily expressed in immune cells; thus, we analyzed the expression patterns of the Padi gene family in T-cells (CD5+ cells), B-cells (CD45R+ cells), macrophages (CD11b + cells), and dendritic cells (MHC class II+ cells). Padi2 and Padi6 were induced in the absence of the Padi4 gene (Fig. 2b, d), and there was no expression of the Padi1 gene in cells of both the wild-type and Padi4^−/−^ mice (data not shown). Padi3 was expressed in these cells but not in those of the Padi4^−/−^ mice (Fig. 2c). These data suggested a compensatory effect among the Padi gene family. In addition, Padi4 was primarily expressed in granulocytes and macrophages (Fig. 2a), a finding consistent with those of previous studies [1, 47]. Furthermore, we isolated and counted the T-cells (CD3+ cells), B-cells (CD19+ cells), NK cells (NK1.1+ cells), macrophages (CD11b + cells), and dendritic cells (MHC class II+ cells) from the spleens of the wild-type mice and Padi4^−/−^ mice using FACS. The numbers of these cells were not significantly different (data not shown).

### Padi4 and Padi2 gene expressions corresponding with CIA treatment

**Fig. 3:**
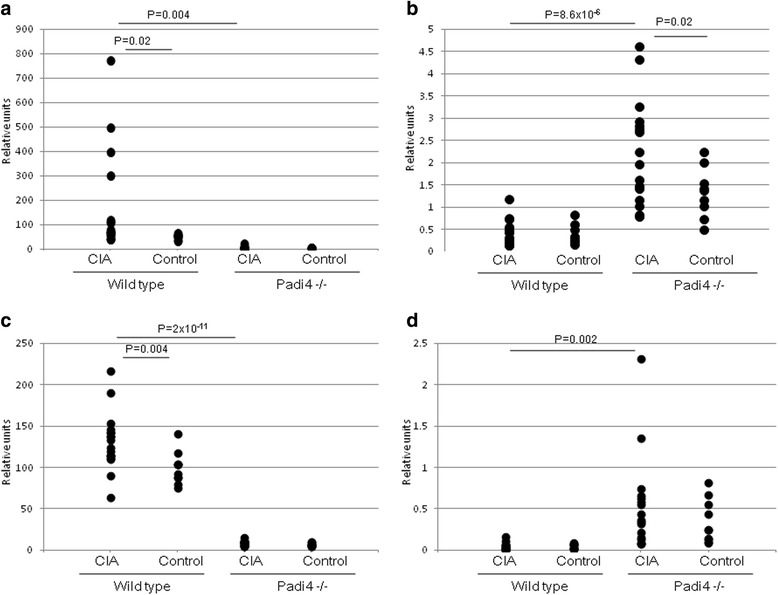
Expression of Padi4 and Padi2 genes in CD11b + and Ly-6G+ cells. Wild-type mice with CIA (*n* = 19), wild-type control mice (*n* = 10), Padi4^−/−^ mice with CIA (*n* = 17), and Padi4^−/−^ control mice (*n* = 10) were used. **a** Padi4 in CD11b + cells, **b** Padi2 in CD11b + cells, **c** Padi4 in Ly-6G+ cells, and **d** Padi2 in Ly-6G+ cells. Relative units were obtained from the comparative ΔΔC_T_ analysis. Statistical analyses were performed using the Student’s *t*-test

Previously, in RA mouse models, although Padi2 mRNA was present in the synovium, it was not translated into the PAD2 protein. In contrast, Padi4 mRNA was absent in the healthy synovium, but it was readily transcribed and translated in neutrophils that infiltrated the synovial tissues during inflammation [48]. To investigate the effects of PAD4 deficiency, we analyzed the expression patterns of Padi2 and Padi4 in macrophages (CD11b + cells) and granulocytes (Ly-6G+ cells) isolated from the splenocytes of the CIA mice to reveal the relationship between Padi2 and Padi4. Padi4 mRNA was not expressed in the macrophages (CD11b + cells) and granulocytes (Ly-6G+ cells) of the control mice, but it was induced by CII immunization (Fig. 3a, c). In the wild-type mice, Padi2 mRNA was not induced by CII immunization, but it was increased by CII immunization in macrophages (CD11b + cells) and granulocytes (Ly-6G+ cells) in the Padi4^−/−^ mice (Fig. 3b, d). These results suggested that Padi2 gene expression was compensationally controlled by Padi4 gene expression.

We investigated PAD2 expression in the PADI4^−/−^ and wild-type mice by fluorescence immunohistochemistry. In macrophages (F4/80+ cells) and NK cells (CD56+ cells), PAD2-expressed cells were increased in the spleens (data not shown). In NK cells, Padi2 RNA had a higher expression in the Padi4^−/−^ mice than in the wild-type mice (data not shown). However, Padi4 and Padi2 were not induced in NK cells during CII immunization (Additional file 4). These data suggested that the induction of Padi2 in NK cells was not related to the development of CIA.

### CIA in the Padi4^−/−^ mice decreases the production of inflammatory cytokines

**Fig. 4:**
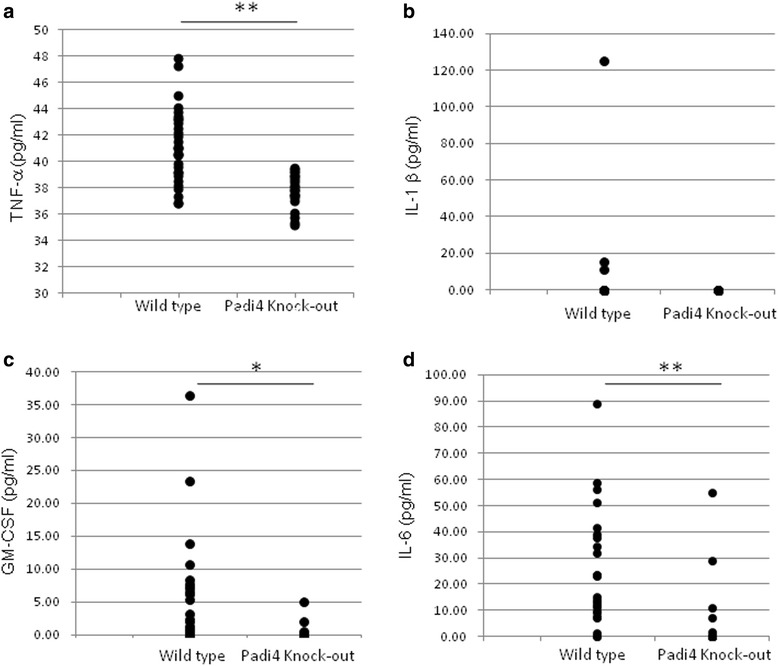
Cytokine response in wild-type and Padi4^−/−^ mice after collagen-induced arthritis (CIA) treatment. Wild-type (*n* = 30) and Padi4^−/−^ (*n* = 24) mice were challenged with CIA, and serum cytokine protein levels were determined by ELISA on day 28. **a** tumor necrosis factor alpha (TNF-α), **b** interleukin (IL)-1β, **c** Granulocyte-macrophage colony-stimulating factor (GM-CSF), **d** IL-6. * *P* < 0.01, **P < 0.001 (Student’s *t*-test)

In patients with RA, activated immune cells release a spectrum of proinflammatory mediators, including TNF-α, IL-6, and IL-1β, which are responsible for joint inflammation and destruction [49]. In particular, TNF-α plays a key pathogenic role. GM-CSF is secreted by T-cells and macrophages following antigen activation, and the function of GM-CSF is indispensable for the growth and development of macrophages. We performed sandwich ELISA to analyze the CIA serum cytokines in the Padi4^−/−^ mice and wild-type mice. In the macrophages/monocytes, inflammatory cytokines such as TNF-α and IL-6 were induced during CIA. TNF-α, IL-6, and GM-CSF in the sera of the PADI4^−/−^ mice showed significant decreases at 28 days (Fig. 4a, c, d). There was no significant difference in IL-1β levels between the Padi4^−/−^ and wild-type mice (Fig. 4b).

**Fig. 5:**
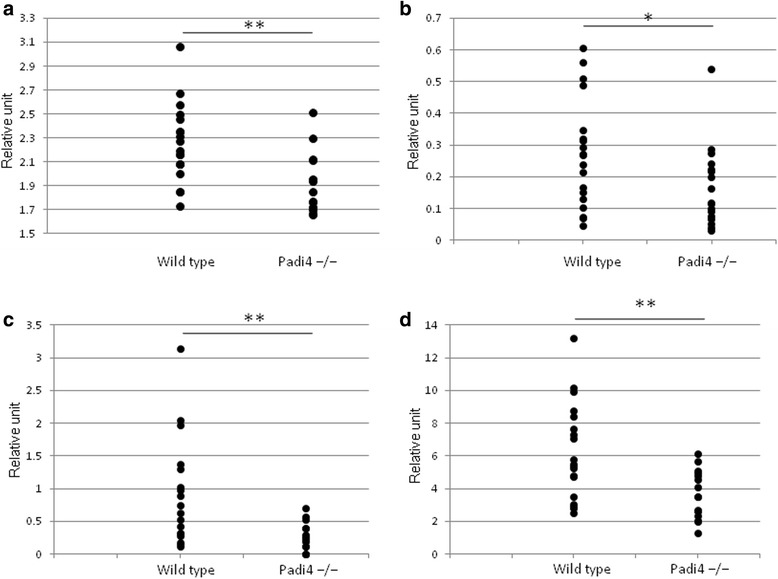
Cytokine mRNA expression in bone marrow derived-macrophages. Bone marrow derived-macrophages were obtained from the bone marrow cells of wild-type (*n* = 19) and Padi4^−/−^ CIA mice (*n* = 17) 10 days after booster injection. The cells were then analyzed by real-time TaqMan RT-PCR for mRNA levels of (**a**) tumor necrosis factor alpha (TNF-α), **b** interleukin (IL)-1β, **c** Granulocyte-macrophage colony-stimulating factor (GM-CSF), and **d** IL-6. Relative units were obtained from the comparative ΔΔC_T_ analysis. * *P* < 0.05, **P < 0.01 (Student’s *t*-test)

Padi4 is primarily expressed in macrophages/monocytes and granulocytes in humans, suggesting that it plays an important role in these cells. In macrophages/monocytes, many key RA cytokines are related, e.g., TNF-α, IL-6, IL-1β, IL-10, matrix metalloproteinase 9 (MMP9), and GM-CSF. We performed expression analysis of cytokines in macrophages using real-time TaqMan RT-PCR assays. In BMMs, the expressions of TNF-α, IL-6, IL-1β, and GM-CSF were significantly higher in the wild-type CIA mice than in the Padi4^−/−^ CIA mice on day 31 (Fig. 5). The expression levels of IL-10 and MMP9 were not significantly different between the wild-type and Padi4^−/−^ CIA mice on day 31 (data not shown). We also evaluated the expression of cytokines by CD11b + cells. The expression of TNF-α, IL-1β, MMP9, and GM-CSF in the wild-type mice was increased and that of IL-10, an anti-inflammatory cytokine, was decreased compared with that in the Padi4^−/−^ mice (Additional file 5).

### Localization of citrullinated proteins in synovial tissues in the CIA mice

**Fig. 6:**
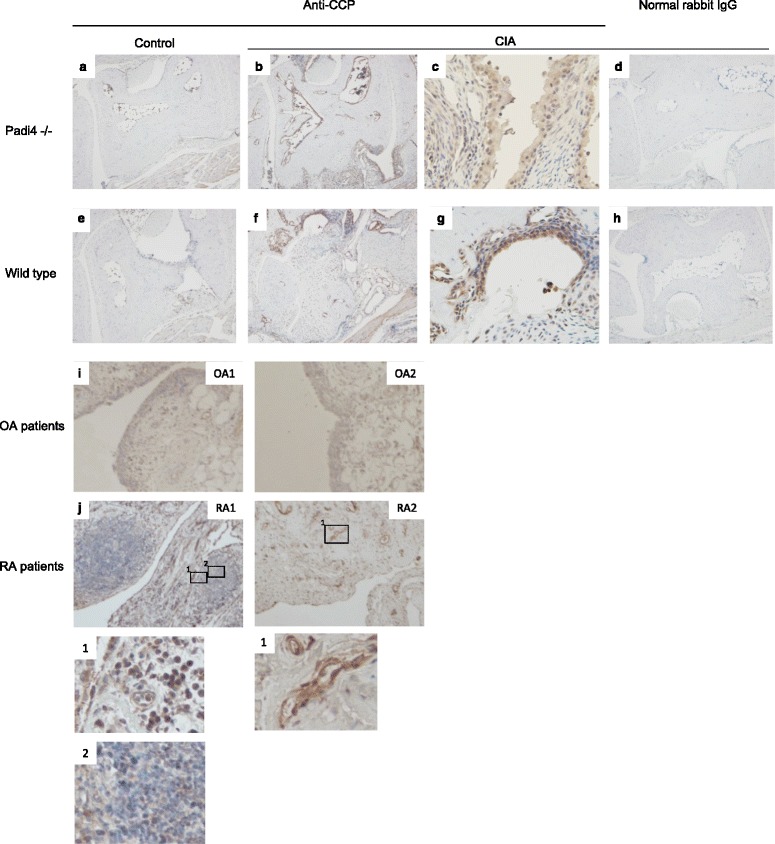
Immunohistochemical localization of antigens detected by anti-CCP antibodies in synovial tissues. Immunohistochemistry with anti-circulated citrullinated peptide (anti-CCP) antibody (Abbiotec), brown staining of the lining and sublining in the inflamed synovial tissue from wild-type collagen-induced arthritis (CIA) mice (**f** and **g**) and Padi4^−/−^ CIA mice (**b** and **c**) and in the synovial tissue from wild-type control mice (**e**) and Padi4^−/−^ control mice (**a**). Staining of a matched irrelevant rabbit IgG-negative control was performed at the same concentration (**d** and **h**). In OA (**i**) and RA synovial tissue samples (**j**), CCP antigens were observed in the synovial sublining and lining layer. Magnification is × 40, except in c, g and j lower (×100)

In patients with RA, ACPAs react with various citrullinated epitopes [50]. The anti-modified citrulline antibody, which has primarily been used for the detection of citrullinated proteins, indicated that there are many citrullinated proteins in RA synovial tissues [51]. However, this antibody cannot identify all synovial citrullinated proteins that bind ACPAs with high affinity and specificity. We investigated whether antigens detected by rabbit anti-CCP antibodies were present in synovial tissues from the CIA mice and patients with RA. Antigens detected by anti-CCP antibodies primarily existed in the lining area of the inflamed synovial tissues from the CIA mice, as shown in Fig. 6. In human RA synovial tissues, antigens of anti-CCP are located in the sublining area as well as the lining layer (Fig. 6). The synovial tissues of some patients with RA had a follicle-like structure (Fig. 6j, RA1), and CCP antigens were observed in the marginal zone of the synovial tissue. The patterns of citrullinated protein localization were the lining layer, the sublining area, and the extracellular matrix.

**Fig. 7:**
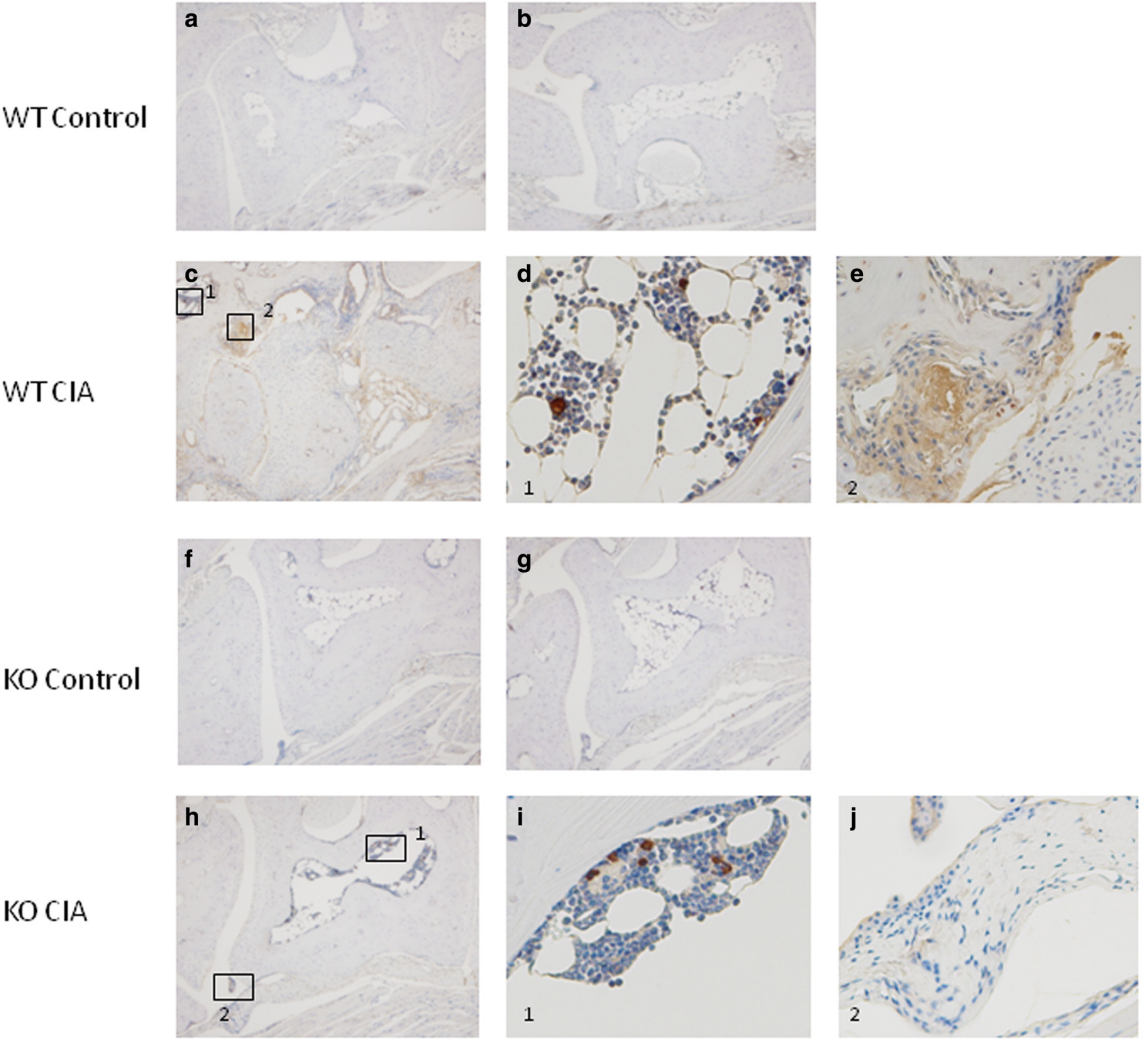
Immunohistochemical localization of citrullinated fibrinogen in synovial tissues of collagen-induced arthritis (CIA) mice. Immunohistochemistry using anti-citrullinated fibrinogen antibody (ModiQuest) and DAB staining of the lining and sublining in an inflamed synovial tissue from wild-type CIA mice (*n* = 3, the scores are 4, c–e) and Padi4^−/−^ CIA mice (*n* = 3, the scores are 4, h–j) and in a synovial tissue from wild-type control mice (*n* = 1, a) and Padi4^−/−^ control mice (*n* = 1, f). Staining of a matched irrelevant rabbit IgG-negative control performed at the same concentration (b and g). (a–c, f–h) Magnification, ×40. (d, e, i, j) Magnification, ×100

Citrullinated fibrinogen is a well-known citrullinated protein and a candidate RA autoantigen. We investigated the distribution of citrullinated fibrinogen in CIA synovium. Citrullinated fibrinogen was observed in the lining layer and sublining area of the wild-type CIA mice (Fig. 7c, e, h, j). However, citrullinated fibrinogen was also localized in the bone marrow cells from both the wild-type and Padi4^−/−^ CIA mice (Fig. 7d, i).

## Discussion

There has been a significant increase in our genetic understanding of RA because of large-scale GWASs that have linked common SNPs to RA risk. A large number of genetic risk factors have been identified in multiple ethnic groups [9, 52, 53]. These genetic risk factors identified through GWASs were found to have modest effect sizes (<1.5). Approximately 30 % of the identified SNPs as causative variants demonstrate cis-eQTL; however, the function of some causative SNPs remains unclear [52].

We previously identified an RA susceptibility variant within PADI4 in a Japanese population. In the case of the PADI4 gene, transcripts generated from the susceptibility haplotype were greater than those generated from the nonsusceptibility haplotype both in vitro and in vivo [1]. A 1.2-fold change in the transcription of PADI4 was observed in peripheral blood leukocytes from individuals with the susceptibility haplotype.

PADI4 produces citrullinated antigens against RA-specific autoantibodies. However, the pathophysiological role of PADI4 remains obscure because a relationship between PADI4 and the development of RA has not been directly demonstrated. Therefore, it is difficult to translate the identification of the susceptibility gene into pathophysiological disease mechanisms.

To reveal the pathophysiological role of the susceptibility genes identified in GWASs, mouse models are powerful tools that allow testing of the in vivo functions of genes to better understand the associations between genes and phenotypes. Some knockout and transgenic mice show varied phenotypes [54, 55].

In the present study, we investigated the nature of the role of PADI4 in CIA and the catalytic function of PADI4 in RA. CIA in the Padi4^−/−^ DBA1J mice was characterized by a significantly decreased arthritis score. After 49 days, the Padi4^−/−^ mice displayed an approximate 50 % disease score compared with the wild-type mice (Fig. 1). Disease incidence was mildly decreased, albeit not significantly (Fig. 1). The average rate of decrease in the disease scores was approximately 25 % (data not shown). Therefore, we found that the Padi4^−/−^ mice showed decreased RA disease scores, consistent with results of the eQTL study by Suzuki et al. [1], and the population attributable risk based on data from their study [1] can be calculated as 24 %. Previously, Rohrbach et al. performed serum-transfer arthritis model with PAD4 KO mice with B6 WT, but there were no statistical significant differences in clinical scores, swelling, joint erosion or joint invasion. In our study, CIA model in B6 strain, there were no statistical significant differences in arthritis scores and was consistent with the previous study. We consider that MHC class II molecule in each strain has a different potential role for autoimmunity [56].

Anti-CII antibodies have been recognized as important pathogenic factors for the development of CIA in mice. In the CIA mice, the Padi4 gene affected the production of anti-CII IgM and IgG (Fig. 2), particularly IgG1 and IgG2a (Additional file 2). The association between elevated IgG2a levels and the occurrence of arthritis indicates the pathogenic role played by Th1 cells and the beneficial role played by Th2 cells [57]. Inflammation in CIA is highly dependent on a predominantly Th1 response, which is characterized by the presence of anti-collagen IgG2a antibodies and the proinflammatory cytokine TNFα. In the Padi4^−/−^ CIA mice sera, anti-CII IgG2a was decreased, suggesting that the deficiency of PAD4 led to the inhibition of Th1-mediated inflammation. This subclass distribution was characteristic of T-cell-dependent antibody production and suggested HLA involvement. These findings may be relevant to the genetic relationship between PADI4 and HLA [16, 58]. The reduction of anti-CII IgM also suggested that Padi4 also have pathologically effect on antigen-specific B cells.

In addition, we measured anti-CCP IgG antibodies using MESACUP ELISA with the CIA mice sera. However, the titers of anti-CCP IgG in the Padi 4^−/−^ CIA mice sera were mildly decreased but not significantly different from those in the wild-type CIA mice sera (data not shown). We also confirmed that citrullinated peptides reacted with CIA sera in the Padi4^−/−^ mice [23, 50, 59, 60]. These data also suggested that the Padi4 gene is compensated for by other Padi gene family members.

We analyzed the expression patterns of the Padi gene family in immune cells isolated from the splenocytes of the wild-type and Padi4^−/−^ mice. In the Padi4^−/−^ mice, the expressions of Padi2 and Padi6 were increased. These data suggested that the deficiency of Padi4 was functionally complemented by Padi2 and Padi6 (Fig. 3). PAD4 only has a nuclear localization signal and the ability to modify histones. It can directly control transcription via histone methylation and citrullination [61]. It is indicated that PADI4 may control the transcription of Padi2 and Padi6. We speculated that the function of Padi4 is important for gene expression via histone citrullination as well as the production of citrullinated autoantigens.

To investigate the function of the Padi4 gene in autoantigen production, we examined the effects of Padi4 on serum cytokine levels. The proinflammatory cytokines TNF-α, IL-1β, and IL-6 were increased at the protein level in the sera of the early-onset CIA mice (Figs. 4, 5). The decrease in the protein levels of Il-1β was not significant whereas those of TNF-α and IL-6 were significantly decreased in the Padi4^−/−^ mice. Moreover, the mRNA levels of these cytokines in the macrophage-derived bone marrow from the Padi4^−/−^ CIA mice were also significantly decreased compared with those in the macrophage-derived bone marrow from the wild-type CIA mice.

GM-CSF also plays a proinflammatory role that has been demonstrated in various models of inflammation and immunity [62]. GM-CSF-deficient mice show decreased susceptibility to CIA; furthermore, anti-GM-CSF is a candidate drug for autoimmune diseases. The protein levels of GM-CSF in sera in the Padi4^−/−^ CIA mice were decreased compared with those in the wild-type CIA mice (Fig. 4, 5). Therefore, Padi4 is involved in inflammation in CIA via effects on proinflammatory cytokines. IL-10 is one of the anti-inflammatory cytokines, and it inhibits the secretion of IL-1β, TNF-α, and IL-6 from macrophages. The expression level of IL-10 in the CD11b + macrophages in the Padi4^−/−^ CIA mice was decreased compared with that in the wild-type CIA mice (Additional file 5).

In a previous report, PAD4 and citrullinated proteins were not detectable before the clinical signs of arthritis; rather, citrullinated proteins and PAD4 increased as the inflammation progressed [63]. These data suggested that PAD4 and citrullinated proteins played important roles in RA development. Although PAD4 has a nuclear localization signal, it is also present in the cytoplasm of infiltrating mononuclear cells [64]. To investigate the localization of citrullinated proteins in the synovial tissues, we performed immunohistochemistry with rabbit anti-CCP antibodies. Antigens recognized by anti-CCP antibodies were observed in joints from both the Padi4^−/−^ and wild-type mice. The antigens were also detected in the lining and sublining areas of proliferated synovial tissues from the wild-type and Padi4^−/−^ CIA mice (Fig. 6). The localization of citrullinated fibrinogen in the synovial tissue exhibited the same pattern as the CCP antigens. We speculated that expressed PAD4 in synovial tissues or synovial fluids play an important role in the supply of citrullinated antigens; however, other PADs should also be involved in the citrullination.

PAD4 regulates transcription via histone methylation. We investigated the effects of PAD4 on the transcriptional pattern of macrophages from the Padi4^−/−^ mice and identified the Fus gene, which was regulated by PAD4 (data not shown). The Fus gene is known as an ALS-related gene and has been implicated in the pathogenesis of myxoid liposarcoma and low-grade fibromyxoid sarcoma [65, 66]. FUS is a member of the TET family and is involved in transcriptional activation. The FUS gene is highly expressed in immune cells such as T-cells, B-cells, monocytes, and NK cells. FUS is a kind of RNA-binding protein and regulated by transcription factor Sp1, GCF, and AP-1. Additionally, our data suggested that Padi4 regulated Fus gene via citrullination of the histone H3. In this study, we also suggested that pathway by which Padi4 and Fus regulate pro-inflammatory cytokines. FUS gene is well-known as related-gene of tumor of human myxoid liposarcoma and associated with amyotrophic lateral sclerosis. However, the pathological function of Fus gene on immune system is not clear.

Recently, it was reported that PADs have various relationships with some diseases such as RA, cancer, Alzheimer’s disease, psoriasis, and colitis [36, 41, 46, 67–69]. The deimination of the PAD substrates may be related with TNF-α signaling. Actually, Shelef et al., indicated that TNF-a induced arthritis had lower levels of autoantibodies using TNF-a-overexpressing and PAD4 deficient mice [70]. Anti-TNF-α monoclonal antibodies are a common treatment for autoimmune diseases (i.e., RA and IBD), suggesting that dysregulated PADs cause uncontrolled TNF-α signaling.

Therefore, PAD enzymes can be novel candidates for the development of a therapy that targets these diseases. Cl-amidine is a bioavailable haloacetamidine-based pan inhibitor that inhibits inflammation in the murine CIA model of RA [40]. It was also reported that Cl-amidine suppressed colitis and breast cancer [41, 69]. However, we could not replicate the inhibition of CIA by Cl-amidine (data not shown). In summary, we demonstrated that PADI4 plays a role in RA progression. Our observations and previous data have indicated that a potential role for dysregulation (i.e., increment) of PAD activity is related to the onset and progression of various diseases, including RA.

## Conclusions

The Padi4 gene, which was identified by large scale multi-ethnic RA GWAS, is functionally related to the RA model mice. In model mice, Padi4 had an effect on arthritis scores but not on the rate of onset. Padi4 had the ability to provide citrullinated antigens and transcriptionally control genes, e.g., inflammatory cytokines. Consequently, inhibitors of PAD4 are good target gene for drug development because there is no physiological abnormality with or without Padi4.

## Additional files

Additional file 1: Generation of a Padi4^−/−^ mouse. (a) Genomic structure of A Padi4^−/−^ mouse. A 5837-bp fragment containing exon 1 and intron 1 of *Padi4* was inserted to the targeting vector. We constructed the targeting vector to replace exon 1 and intron 1, including the transcription initiation site, using mouse PGK-1 promoter and the neomycin-resistance gene. We confirmed the knockout condition by Southern blotting (b) and RT-PCR (c). (d) Distributions of immune cells in the spleen of wild-type (*n* = 3) and Padi4^−/−^ (*n* = 3) mice were analyzed by FACS. (e) Hematoxylin and eosin (HE) staining of Padi4-expressed tissues from the wild-type (WT) mouse, Padi4^+/−^ (Heterozygote) mouse, and Padi4^−/−^ (KO) mouse. (PDF 458 KB) Additional file 2: Serum anti-CII antibodies in CIA using Wild type and Padi4−/− mice. Anti-CII IgG1 antibodies (a) and IgG2a antibodies (b) in the sera of 31 Wild type CIA mice, 14 Wild type control mice, 28 Padi4−/− CIA mice, and 14 Padi4−/– control mice at day 35 after collagen injection. *P < 0.01 (Student’s *t*-test). (TIF 292 KB) Additional file 3: Padi2 and Padi4 are expressed in the spleens of wild-type mice. (a–e) Immunofluorescence staining (×40) shows that Padi2 is expressed ubiquitously in the spleen. Spleens were probed with anti-Padi2 (green fluorescent signal) and cell surface markers (red fluorescence signal; a, CD3; b, B220; c, Gr-1; d, CD56; e, F4/80). The nuclei were stained with DAPI (blue fluorescence signal). The arrows indicate the colocalization of Padi2 and each cell surface marker. (f–k) Immunofluorescence staining (×40) shows that Padi4 was expressed in splenocytes. The spleens were probed with anti-Padi4 (green fluorescence signal), and cell surface markers (red fluorescence signal; f, CD3; g, B220; h, Gr-1; i, CD56; j, F4/80). Nuclei were stained with DAPI (blue fluorescence signal). (K) Immunofluorescence staining (left, ×40; right, ×280) shows that Padi4 was localized in both the nuclei and cytoplasm of macrophages, which expressed F4/80. (PDF 286 KB) Additional file 4: Padi2 and Padi4 expression in NK1.1+ cells. mRNA levels were determined by real-time TaqMan RT-PCR using NK1.1+ cells as a reference for GAPDH normalization. Padi2 and Padi4 mRNA expressions were not different between wild-type collagen-induced arthritis (CIA) (n = 10) and control (n = 8) mice spleens. (PDF 209 KB) Additional file 5: Cytokine mRNA expression in CD11b + macrophages in the spleen. CD11b + wild-type (n = 19) and Padi4^−/−^ CIA mice (n = 17) were used for 10 days from the day after booster injection. The cells were analyzed by real-time TaqMan RT-PCR for mRNA levels of (a) tumor necrosis factor alpha (TNF-α), (b) CSF-2, (c) IL-1β, (d) IL-6, and (e) IL-10. **P* < 0.05, ***P* < 0.01 (Student’s *t*-test). (PDF 173 KB)

## Abbreviations

Actb: beta-actin
BSA: Bovine serum albumin
CT: Threshold cycle
CD: clusters of differentiation
DAPI: 4’, 6-diamidino-2-phenylindole
EDTA: Ethylenediaminetetraacetic acid
FBS: Fetal bovine serum
GM-CSF: Granulocyte-macrophage colony-stimulating factor
IL: interleukin
Ig: immunoglobulin
LPS: lipopolysaccharide
MHC: major histocompatibility complex
M-CSF: macrophage colony-stimulating factor
MMP: matrix metalloproteinase
RT-PCR: reverse transcription-polymerase chain reaction
TNF-α: tumor necrosis factor alpha
ΔΔCT: Delta-delta CT
