# Supplementary figures and images for: Decreased severity of experimental autoimmune arthritis in peptidylarginine deiminase type 4 knockout mice
Source: BMC Musculoskelet Disord. 2016 May 5;17:205. doi: 10.1186/s12891-016-1055-2 (PMC4858923; doi:10.1186/s12891-016-1055-2)

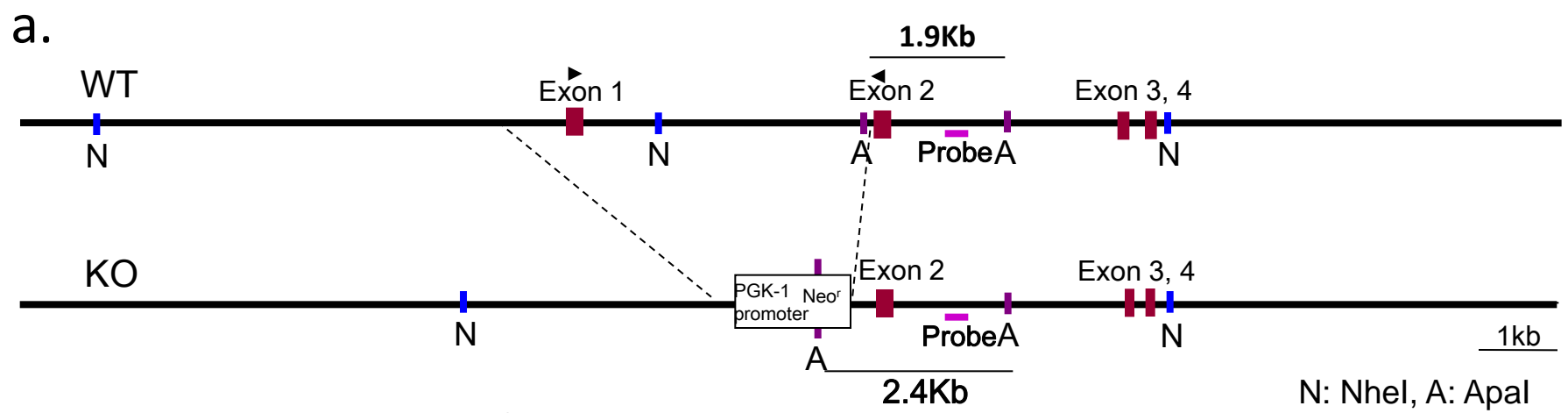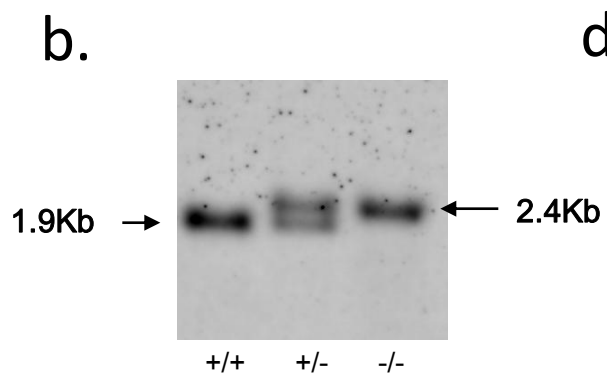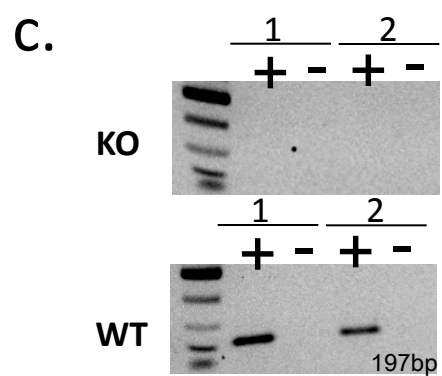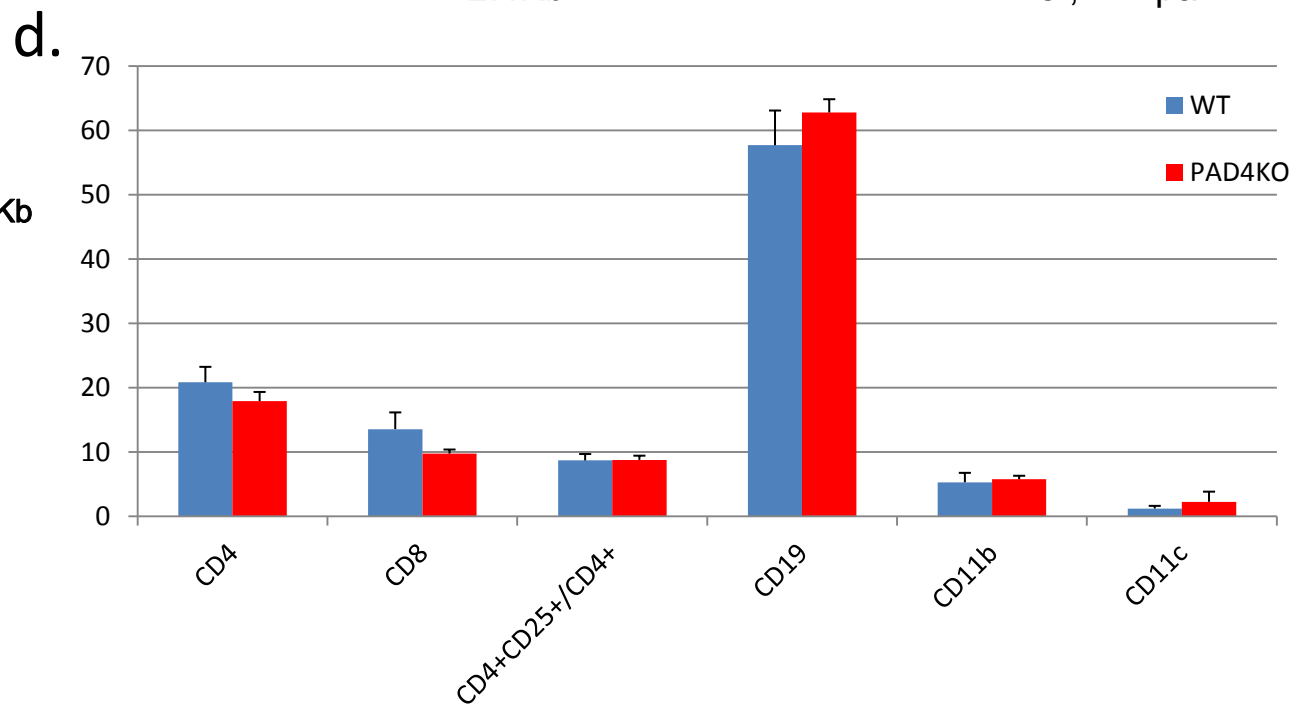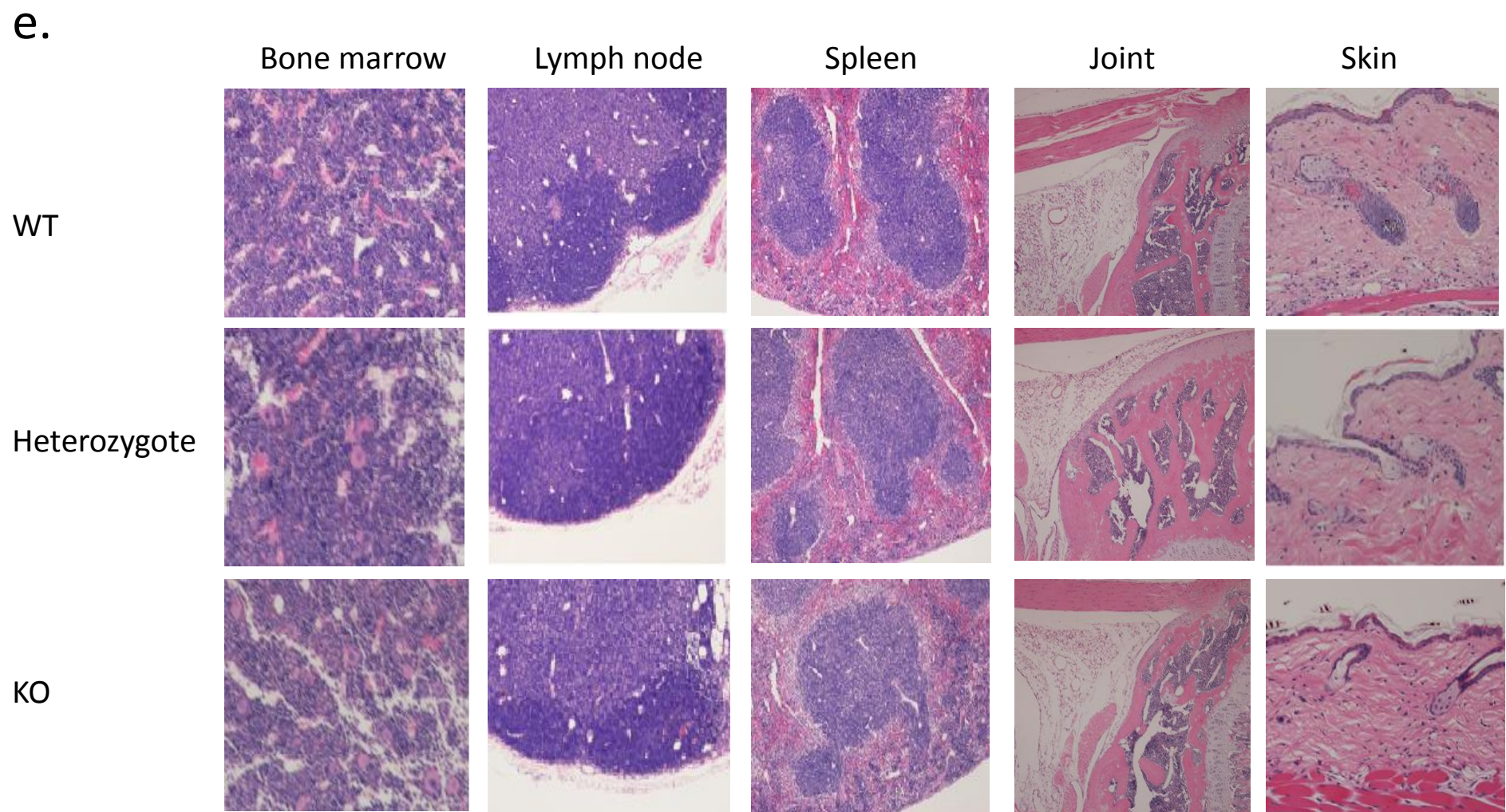

Supplement: Additional file 1: — Generation of a Padi4−/− mouse. (a) Genomic structure of A Padi4−/− mouse. A 5837-bp fragment containing exon 1 and intron 1 of Padi4 was inserted to the targeting vector. We constructed the targeting vector to replace exon 1 and intron 1, including the transcription initiation site, using mouse PGK-1 promoter and the neomycin-resistance gene. We confirmed the knockout condition by Southern blotting (b) and RT-PCR (c). (d) Distributions of immune cells in the spleen of wild-type (n = 3) and Padi4−/− (n = 3) mice were analyzed by FACS. (e) Hematoxylin and eosin (HE) staining of Padi4-expressed tissues from the wild-type (WT) mouse, Padi4+/− (Heterozygote) mouse, and Padi4−/− (KO) mouse. (PDF 458 KB) [file 12891_2016_1055_MOESM1_ESM.pdf]

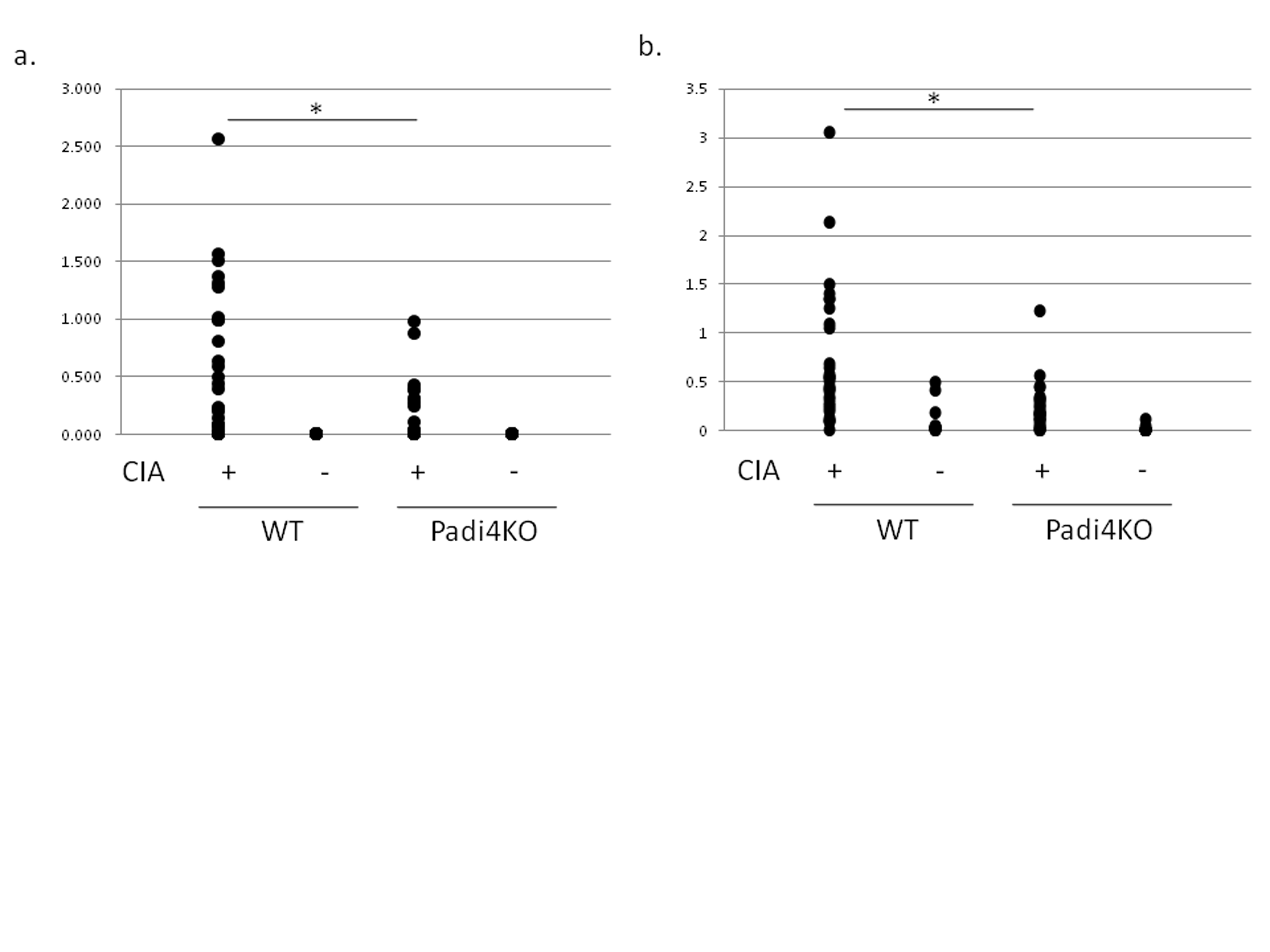

Supplement: Additional file 2: — Serum anti-CII antibodies in CIA using Wild type and Padi4−/− mice. Anti-CII IgG1 antibodies (a) and IgG2a antibodies (b) in the sera of 31 Wild type CIA mice, 14 Wild type control mice, 28 Padi4−/− CIA mice, and 14 Padi4−/– control mice at day 35 after collagen injection. *P < 0.01 (Student’s t-test). (TIF 292 KB) [file 12891_2016_1055_MOESM2_ESM.tif]

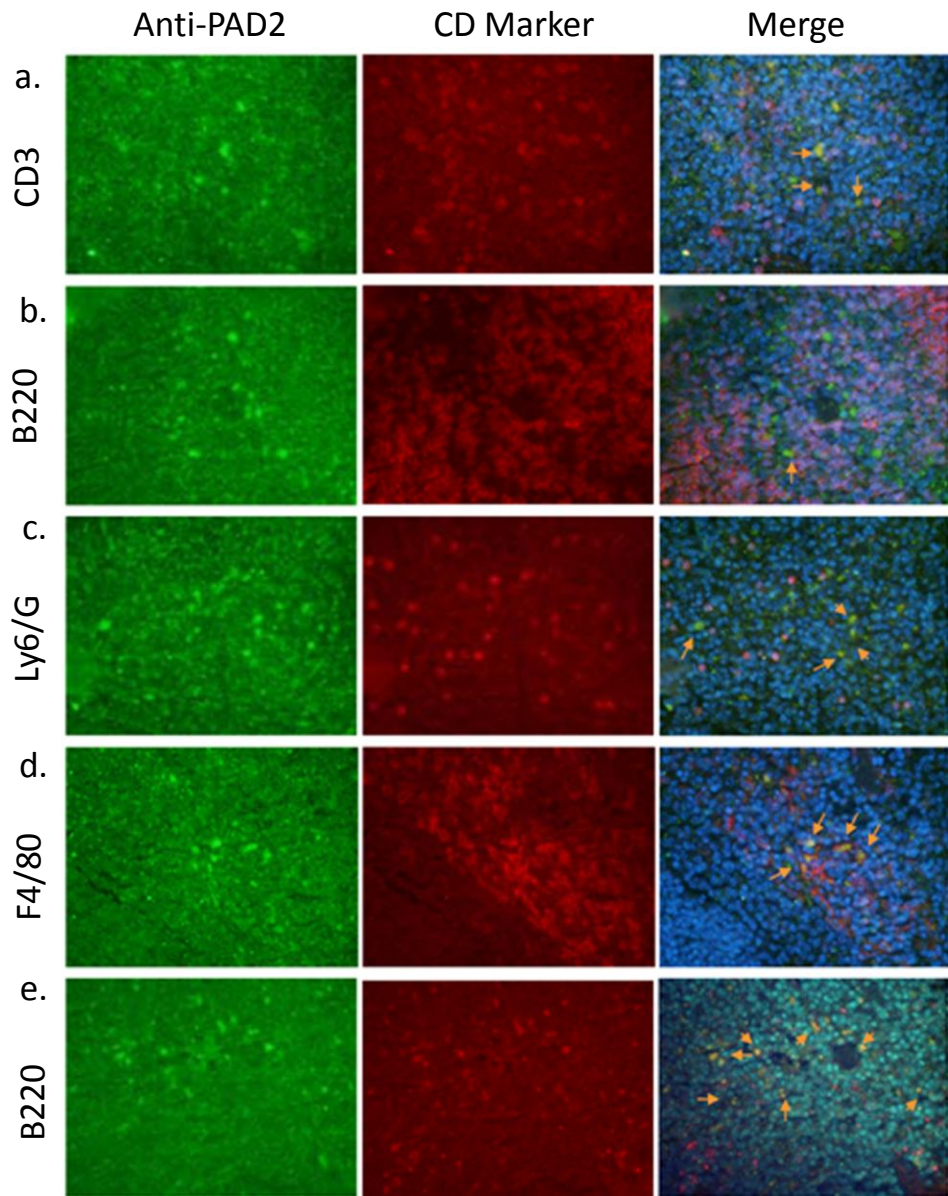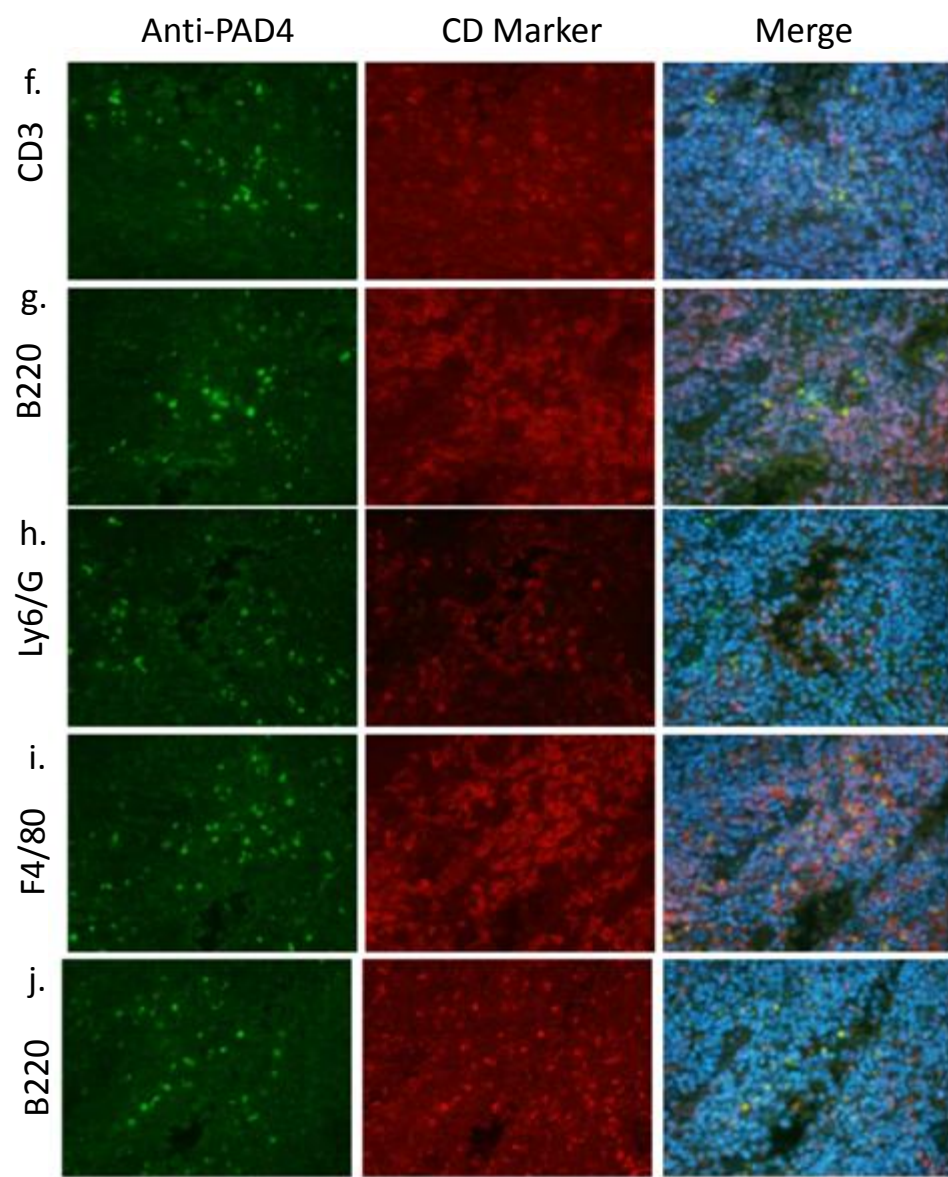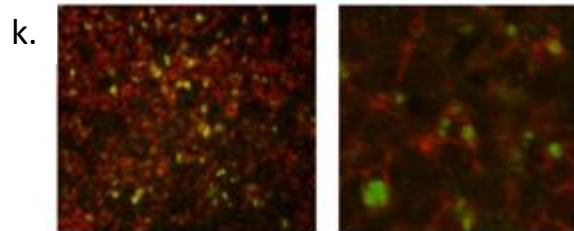

Supplement: Additional file 3: — Padi2 and Padi4 are expressed in the spleens of wild-type mice. (a–e) Immunofluorescence staining (×40) shows that Padi2 is expressed ubiquitously in the spleen. Spleens were probed with anti-Padi2 (green fluorescent signal) and cell surface markers (red fluorescence signal; a, CD3; b, B220; c, Gr-1; d, CD56; e, F4/80). The nuclei were stained with DAPI (blue fluorescence signal). The arrows indicate the colocalization of Padi2 and each cell surface marker. (f–k) Immunofluorescence staining (×40) shows that Padi4 was expressed in splenocytes. The spleens were probed with anti-Padi4 (green fluorescence signal), and cell surface markers (red fluorescence signal; f, CD3; g, B220; h, Gr-1; i, CD56; j, F4/80). Nuclei were stained with DAPI (blue fluorescence signal). (K) Immunofluorescence staining (left, ×40; right, ×280) shows that Padi4 was localized in both the nuclei and cytoplasm of macrophages, which expressed F4/80. (PDF 286 KB) [file 12891_2016_1055_MOESM3_ESM.pdf]

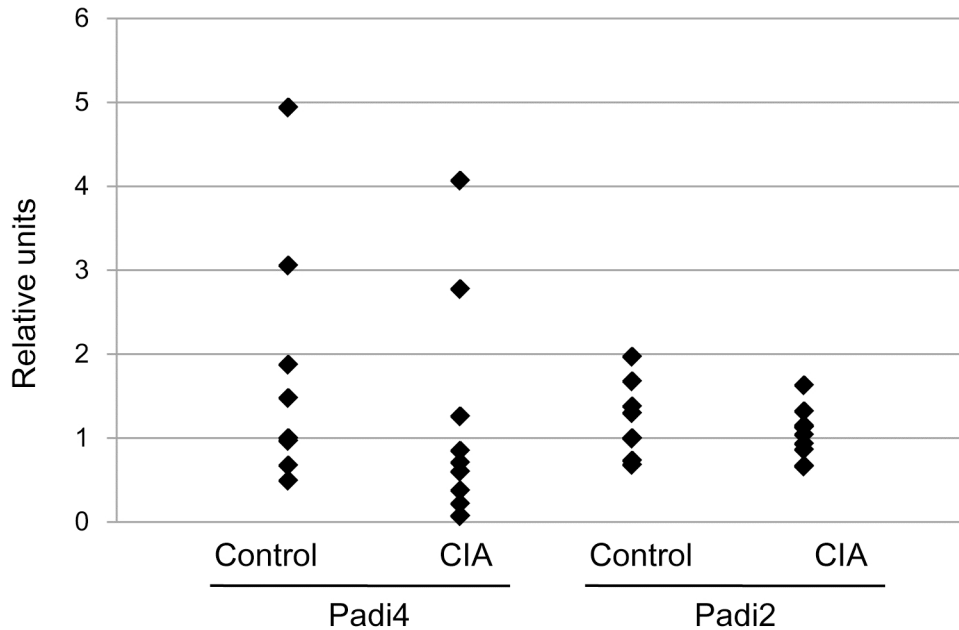

Supplement: Additional file 4: — Padi2 and Padi4 expression in NK1.1+ cells. mRNA levels were determined by real-time TaqMan RT-PCR using NK1.1+ cells as a reference for GAPDH normalization. Padi2 and Padi4 mRNA expressions were not different between wild-type collagen-induced arthritis (CIA) (n = 10) and control (n = 8) mice spleens. (PDF 209 KB) [file 12891_2016_1055_MOESM4_ESM.pdf]

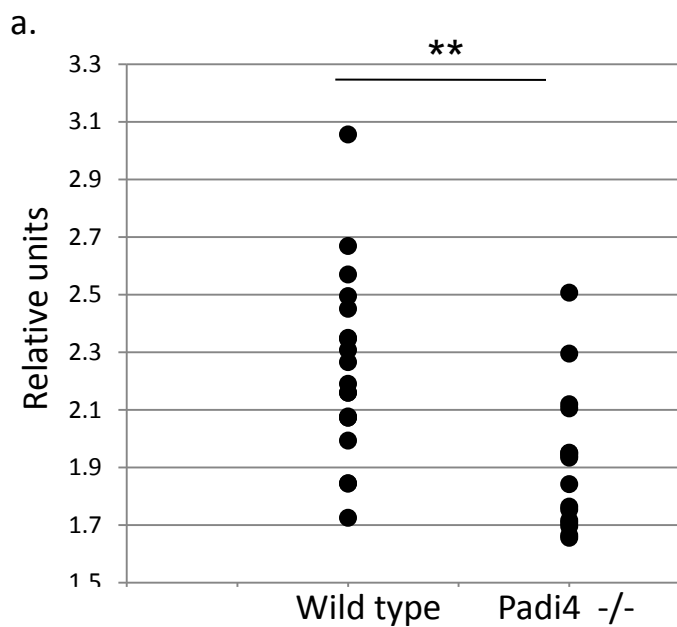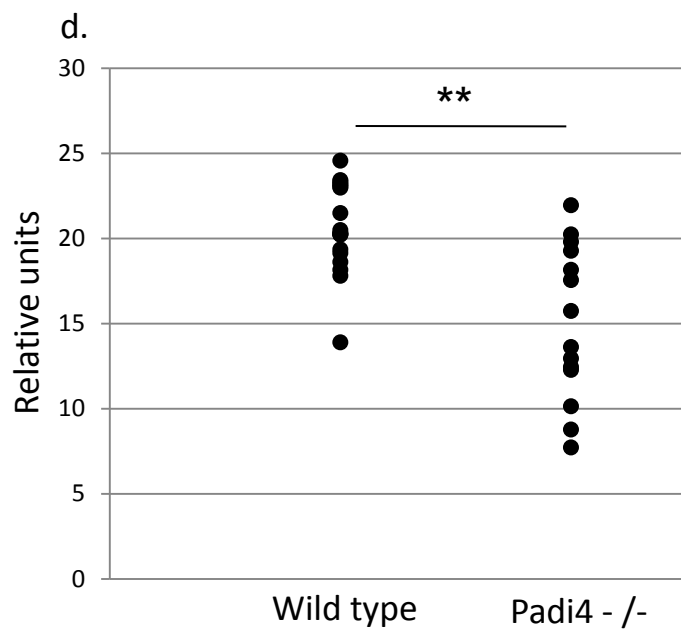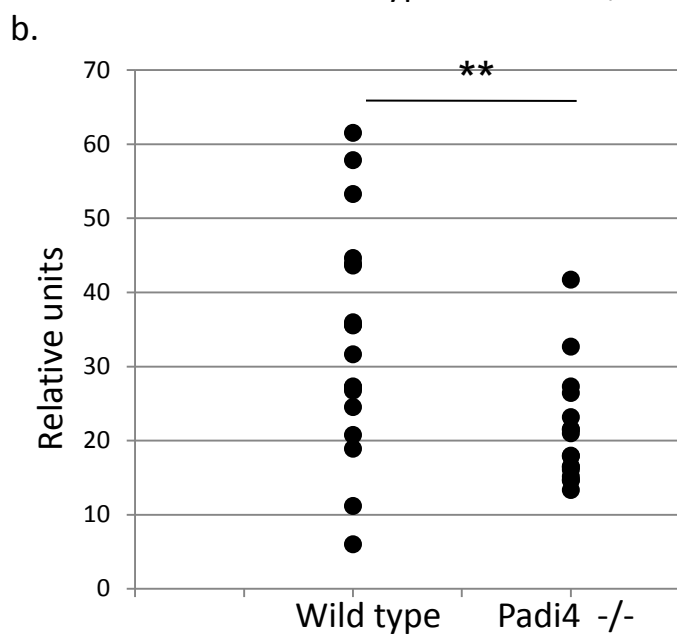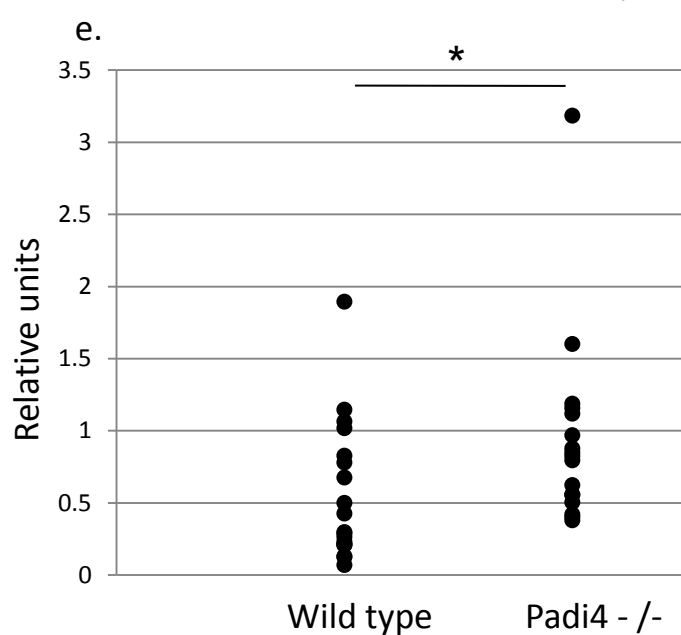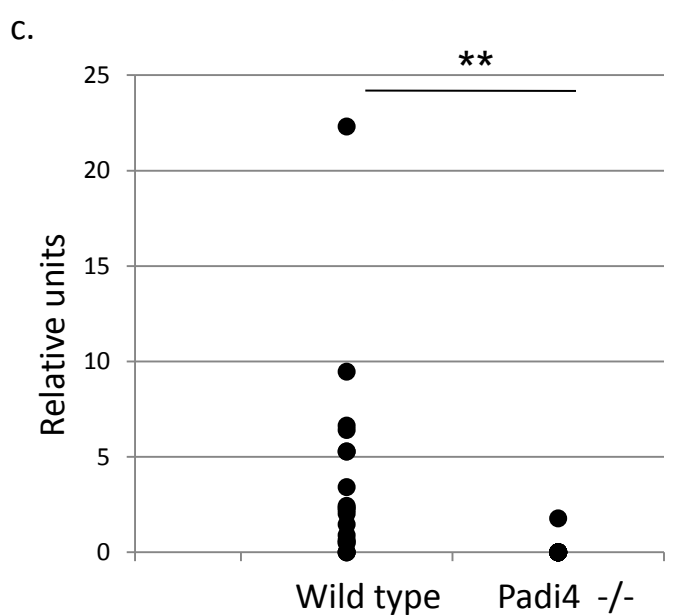

Supplement: Additional file 5: — Cytokine mRNA expression in CD11b + macrophages in the spleen. CD11b + wild-type (n = 19) and Padi4−/− CIA mice (n = 17) were used for 10 days from the day after booster injection. The cells were analyzed by real-time TaqMan RT-PCR for mRNA levels of (a) tumor necrosis factor alpha (TNF-α), (b) CSF-2, (c) IL-1β, (d) IL-6, and (e) IL-10. *P < 0.05, **P < 0.01 (Student’s t-test). (PDF 173 KB) [file 12891_2016_1055_MOESM5_ESM.pdf]
